# Towards greener reduced graphene oxide: a critical review of environmentally driven reduction strategies

**DOI:** 10.1039/d5ra08914j

**Published:** 2026-01-07

**Authors:** Md. Saiful Islam Monir, Abdur Rahman, Prianka Saha, Ismail Rahman, Md. Mahiuddin

**Affiliations:** a Chemistry Discipline, Khulna University Khulna 9208 Bangladesh mahiuddin@chem.ku.ac.bd; b Institute of Environmental Radioactivity, Fukushima University 1 Kanayagawa Fukushima-Shi Fukushima 960-1296 Japan immrahman@ipc.fukushima-u.ac.jp

## Abstract

The synthesis of graphene-based materials has attracted immense interest due to their exceptional properties. However, graphene oxide (GO), a common precursor, contains oxygen-containing functional groups that disrupt its sp^2^ carbon network, thereby limiting its electrical conductivity and other key properties. The reduction of GO to reduced graphene oxide (rGO) is therefore a crucial step in restoring these properties. Traditional reduction methods often use toxic, hazardous chemical reagents, such as hydrazine, which pose significant environmental and health risks. Consequently, there is a pressing need for environmentally benign, sustainable, and cost-effective reduction strategies. This review provides a critical examination of green reduction methods for GO, focusing on plant extracts, microorganisms, and isolated biomolecules as sustainable reducing agents. It moves beyond a simple summary of existing literature to offer a comparative analysis of these methods, evaluating their reduction efficacy based on key material properties, such as the C/O ratio, electrical conductivity, and structural integrity, as determined by spectroscopic and microscopic techniques (UV-vis, XRD, Raman, XPS, SEM, TEM). The central focus of this review is to establish a clear link between the choice of green reduction strategy, the resulting physicochemical properties of the rGO, and its performance in specific technological applications, including energy storage, sensing, environmental remediation, and biomedicine. By analyzing reaction mechanisms, scalability, and application-specific outcomes, this review identifies current research gaps and provides a forward-looking perspective on the rational design of green-synthesized rGO for advanced, sustainable technologies.

## Abstract 2

The green synthesis of reduced graphene oxide has gained considerable attention due to its environmentally friendly nature and the retention of its exceptional physicochemical properties and wide-ranging applications.

## Graphical abstract

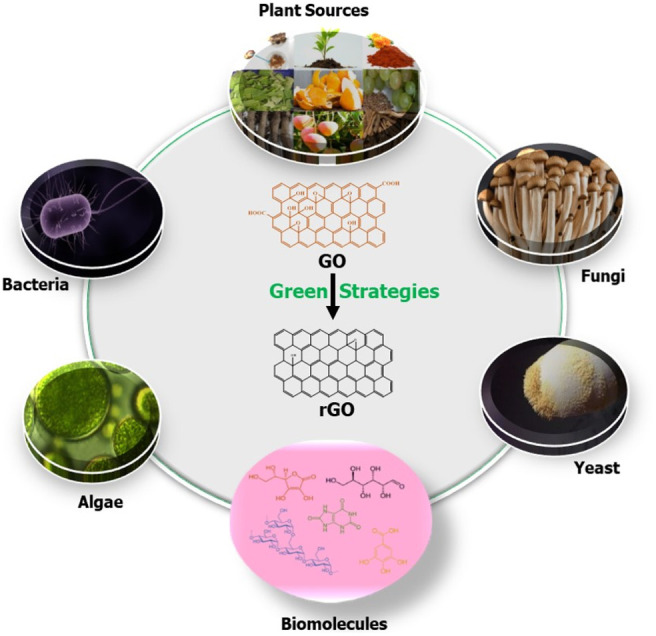

## 1 Introduction

**Fig. 1:**
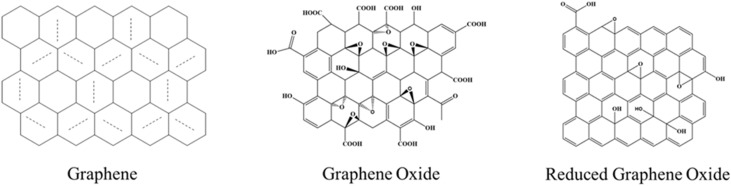
Structures of graphene (G), graphene oxide (GO), and reduced graphene oxide (rGO), illustrating the removal of oxygen functional groups during reduction.

Graphene, a two-dimensional monolayer of sp^2^-hybridized carbon atoms arranged in a hexagonal lattice, was first isolated in 2004, marking the beginning of a new era in materials science.^1^ Its extraordinary electrical, thermal, mechanical, and optical properties have positioned it as a transformative material for applications in electronics, drug delivery, sensors, and energy storage.^2–6^ One of the most viable routes to large-scale production of graphene-based materials is chemical exfoliation of graphite. This top-down approach, however, yields graphene oxide (GO) rather than pristine graphene.^7–10^ GO is a graphene sheet decorated with oxygen-containing functional groups, primarily hydroxyl and epoxide groups on its basal plane, and carboxyl and carbonyl groups at its edges (Fig. 1).^11^

The synthesis of GO is typically achieved through strong oxidation of graphite, with methods developed by Brodie, Staudenmaier, and Hummers being foundational.^12–15^ The modified Hummers' method is now widely used as it improves safety and efficiency.^16^ While the oxygen functional groups render GO hydrophilic and dispersible, they disrupt the π-conjugated network, making it electrically insulating and unsuitable for many applications. To restore the graphene-like properties, GO must be reduced to rGO. The primary goal of this reduction is to remove the oxygen-containing groups, thereby recovering the sp^2^-conjugated structure and significantly enhancing electrical and thermal conductivity.^17^ The effectiveness of any reduction process is typically quantified by the increase in the carbon-to-oxygen (C/O) ratio and the corresponding improvement in electrical conductivity.^18^

**Fig. 2:**
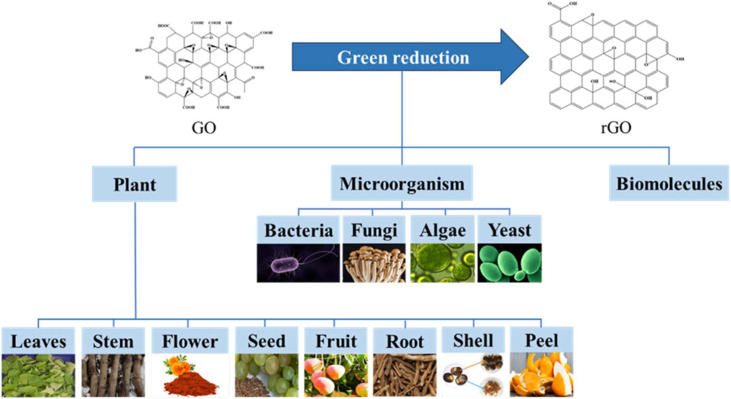
Classification of green reductants for the reduction of GO into three main categories: plants, microorganisms, and biomolecules, with further subdivisions.

Conventional reduction methods often rely on highly toxic and hazardous chemicals, such as hydrazine hydrate and sodium borohydride, or on energy-intensive thermal annealing processes.^19,20^ These approaches raise significant environmental and safety concerns. In response, the field has shifted towards “green” reduction strategies that utilize non-toxic, sustainable, and cost-effective reducing agents derived from natural sources. As illustrated in Fig. 2, these green reductants can be broadly classified into three categories: plant extracts, microorganisms (*e.g.*, bacteria, fungi, yeast), and isolated biomolecules (*e.g.*, vitamins, amino acids, sugars).^21–24^ The phytochemicals, enzymes, and other bioactive compounds within these sources not only reduce GO but often act as stabilizing agents, preventing the agglomeration of rGO sheets.^25^

While several works have explored the green synthesis of rGO, they have served mainly as summaries of published reports. This review aims to provide a more critical and analytical perspective. This work not only surveys the various green reduction strategies but also critically evaluates their efficacy by directly linking the choice of reductant and reaction conditions to the final material properties. By integrating discussions of synthesis, characterization, and application, this review aims to establish a clearer understanding of the structure–property–application relationships for green-synthesized rGO. The objective is to move beyond mere cataloging and provide deeper insights into mechanistic understanding, identify specific research gaps, and offer a forward-looking perspective to guide the rational design of rGO for targeted, high-impact applications.

## 2 Fundamental properties of GO and rGO: the motivation for reduction

The transformation from GO to rGO involves a fundamental change in material properties, driven by the removal of oxygen functional groups and the restoration of the sp^2^ carbon lattice. Understanding these changes is crucial to appreciating the importance of the reduction process.

### 2.1 Electrical conductivity

Pristine graphene is a semi-metal with an electrical conductivity that can reach up to 2000 S cm^−1^.^26^ In contrast, GO is an electrical insulator, with sheet resistance values often exceeding 10^12^ Ω sq^−1^ and conductivity as low as 0.0486 S m^−1^.^27–29^ The insulating nature is a direct consequence of the sp^3^ C–O bonds that disrupt the percolating pathways for charge carriers among the remaining sp^2^ carbon clusters. The reduction process systematically removes these sp^3^ defects, restoring the conjugated sp^2^ network and dramatically increasing electrical conductivity. The final conductivity of rGO is highly dependent on the degree of reduction. For instance, rGO synthesized using gallic acid exhibits a modest conductivity of 0.358 S m^−1^,^30^ whereas microbial reduction with *Shewanella oneidensis* yields a conductivity of 55.32 S m^−1^.^31^ While these values represent a significant improvement over GO, they remain lower than those of rGO produced *via* hazardous chemical reductants, such as hydrazine (216.56 S m^−1^),^31^ highlighting the ongoing challenge of achieving complete reduction using green methods.

### 2.2 Thermal conductivity

Graphene possesses an exceptionally high thermal conductivity (4840–5300 W m^−1^ K^−1^), making it ideal for thermal management applications.^32^ The oxidation process introduces structural defects and vacancies that act as phonon scattering sites, causing the thermal conductivity of GO to plummet to around 2.90 W m^−1^ K^−1^. The reduction to rGO partially restores the lattice integrity, leading to a significant recovery in thermal conductivity. For example, an rGO film produced using dopamine as a reductant exhibited a thermal conductivity of 13.42 W m^−1^ K^−1^,^33^ and values as high as 500 W m^−1^ K^−1^ have been reported for well-reduced, annealed films.^34^

### 2.3 Mechanical strength

While GO possesses impressive mechanical strength, it is inherently lower than that of pristine graphene due to the structural defects introduced by the oxygen functional groups. The reduction process, by restoring the sp^2^-conjugated structure, generally enhances mechanical properties. The Young's modulus of an rGO film can be significantly higher than that of a GO film. For example, one study reported that the Young's modulus increased from 27.3 GPa for an unannealed rGO film to 158.0 GPa after annealing, demonstrating the improved rigidity that comes with a more ordered, graphene-like structure.^35^

### 2.4 Chemical stability and dispersibility

The abundant oxygen functional groups on GO make it highly reactive and hydrophilic, allowing it to form stable colloidal dispersions in water. This property is advantageous for solution-based processing. However, upon reduction, the removal of these polar groups restores the hydrophobic nature of the carbon lattice. Consequently, rGO sheets have a strong tendency to agglomerate and restack in aqueous solutions due to π–π interactions and van der Waals forces, making them less dispersible.^36,37^ A key advantage of many green reduction methods is that the phytochemicals or biomolecules used for reduction often co-functionalize the rGO surface, acting as stabilizing or capping agents that improve dispersibility without compromising the restored electronic properties.

### 2.5 Surface area

Theoretically, GO can have a surface area as high as 736.6 m^2^ g^−1^.^38^ However, in practice, the wrinkling and distortion of the sheets caused by functional groups lead to much lower values (*e.g.*, 18 m^2^ g^−1^).^17^ The reduction process typically increases the specific surface area by removing the bulky oxygen groups and creating a more porous, accessible structure. The final surface area is highly dependent on the reductant. rGO produced with lemon juice showed a surface area of 159 m^2^ g^−1^,^17^ while microbial reduction with *Shewanella* achieved 243.24 m^2^ g^−1^.^39^ These values, while substantial, are often lower than those achieved with chemical reductants like hydrazine (400–700 m^2^ g^−1^),^40^ indicating that green methods can sometimes leave behind residues or cause more sheet aggregation, affecting the final morphology.

## 3 Green reduction strategies: a critical and integrated analysis

A critical analysis of the three primary green reduction strategies is provided in this section. For each method, the general methodology is discussed, the reduction efficacy is evaluated by synthesizing data from the literature, the proposed chemical mechanisms are compared, and direct links are made between the resulting material properties and their performance in specific, high-value applications.

### 3.1 Reduction of GO using plant extracts

Plant extracts have emerged as one of the most popular choices for green GO reduction due to their low cost, ready availability, and rich composition of phytochemicals, including polyphenols (flavonoids, tannins), alkaloids, and vitamins, which can act as potent reducing agents.^41–44^

#### 3.1.1 General methodology and reduction efficacy

**Fig. 3:**
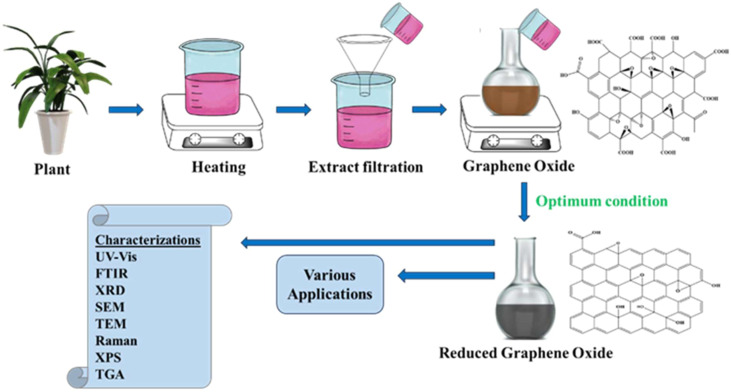
Schematic diagram illustrating the typical process for the green reduction of GO using plant extracts, followed by characterization and application.

The typical procedure involves preparing an aqueous extract from a specific plant part (leaves, roots, fruit, *etc.*), mixing it with a GO dispersion, and heating the mixture, often under reflux, until the color changes from brown to black, signifying the formation of rGO (Fig. 3).^45,46^ The resulting rGO is then washed and dried.

A critical evaluation of the extensive literature reveals a wide range of reduction efficacy depending on the plant source and reaction conditions. A meta-analysis of the studies compiled in Table 1 shows several key trends. Firstly, extracts derived from sources known for high concentrations of potent antioxidants, such as artemisinin and wild carrot root, achieve exceptionally high C/O ratios of 11.7 and 11.9, respectively.^47,48^ These values are superior to many other green methods and are competitive with the C/O ratio of 9.82 obtained using hydrazine.^49^ This suggests that the specific chemical nature of the reductant is more critical than its general classification as a “phytochemical.” Secondly, reaction conditions play a crucial role; methods that employ heating or reflux consistently outperform those conducted at room temperature, indicating that thermal energy is necessary to overcome the activation barrier for deoxygenation. For instance, rGO produced from banana peel extract under reflux yielded a C/O ratio of 3.80, a value significantly better than those reported in many room-temperature syntheses.^50^ However, the complexity of plant extracts, which contain dozens of compounds, leads to a lack of selectivity in functionalization and can result in batch-to-batch variability, posing a challenge for scalable and reproducible manufacturing.

**Table 1:** Summary of plant extracts utilized in the reduction of GO, with key characterization data^a^

Plants/plant extracts	Part used	Reduction conditions	Characteristics	Application	Ref.
*Camellia oleifera*	Shell	Water bath, 80 °C for 3 h	2*θ*: 24.9°, *I*_D_/*I*_G_ (rGO): 1.01 > *I*_D_/*I*_G_ (GO): 0.90	Adsorption of copper(ii)	51
Carrot	Root	Stir, 48 h at 150 rpm and reflux, 100 °C for 24 h	2*θ*: 24.2°, C/O ratio: 3.91, *I*_D_/*I*_G_ (rGO): 0.94 > *I*_D_/*I*_G_ (GO): 0.83	Supercapacitor	52
Lemon juice	Fruit	Stir, 24 h at 150 rpm and reflux, 100 °C for 8 h	2*θ*: 25.1°, C/O ratio: 4.91, *I*_D_/*I*_G_ (rGO): 0.96 > *I*_D_/*I*_G_ (GO): 0.83	Supercapacitor	52
*Persea americana*	Seed	Stir, 100 °C for 10 h	UV-vis peak: 280 nm, *I*_D_/*I*_G_ (rGO): 0.89 > *I*_D_/*I*_G_ (GO): 0.76	Antibacterial activity	53
*Plectranthus amboinicus*	Leaves	Autoclave, 100 °C for 12 h	2*θ*: 25°, *I*_D_/*I*_G_ (rGO): 1.297 > *I*_D_/*I*_G_ (GO): 1.07	Supercapacitor	20
*Cinnamomum zeylanicum*	Bark	Reflux, 45 min	UV-vis peak: 280 nm, 2*θ*: 23°	Dye elimination and antioxidant activity	54
*Tithonia diversifolia*	Flower	Stir, 80 °C for 12 h	UV-vis peak: 265 nm, 2*θ*: 24°–26°	Cytotoxicity	55
*Lantana camara*	Leaves	Reflux, 24 h	UV-vis peak: 273 nm, 2*θ*: 21.9°, *I*_D_/*I*_G_ (rGO): 0.37 < *I*_D_/*I*_G_ (GO): 0.98	Antibacterial, antioxidant and cytotoxicity activity	25
*Phyllanthus emblica*	Fruit	Reflux, 95 °C for 3 h	UV-vis peak: 270 nm, 2*θ*: 23.11°, *I*_D_/*I*_G_ (rGO): 1.11 < *I*_D_/*I*_G_ (GO): 1.29	Photovoltaic activity	56
Banana	Peel	Reflux, 90 °C for 48 h	C/O ratio: 81.0 : 19.0, *I*_2D_/*I*_G_ (rGO): 0.84117 > *I*_2D_/*I*_G_ (GO): 0.00005	n.d.	45
Banana	Fruit	Reflux, 90 °C for 48 h	C/O ratio: 78.1 : 21.9, *I*_2D_/*I*_G_ (rGO): 0.42773 > *I*_2D_/*I*_G_ (GO): 0.00005	n.d.	45
*Caesalpinia sappan* L.	Flower	Autoclave, 100 °C for 6 h	UV-vis peak: 259 nm, 2*θ*: 25.67°, *I*_D_/*I*_G_ (rGO): 1.29 < *I*_D_/*I*_G_ (GO): 1.60	n.d.	57
Artemisinin	Leaves	Water bath, 95 °C for 24 h	C/O ratio: 11.7, *I*_D_/*I*_G_ (rGO): 1.32 > *I*_D_/*I*_G_ (GO): 0.90	n.d.	47
*Chenopodium album*	Vegetable	Reflux, 100 °C for 12 h	UV-vis peak: 263 nm, 2*θ*: 22.50°	Antimicrobial and anticancer activity	58
Eucalyptus	Leaves	Stir, 80 °C for 8 h	UV-vis peak: 273.5 nm	Dye removal	59
Lemon juice	Fruit	Reflux, RT for 45 min	UV-vis peak: 259 nm, 2*θ*: 30°	Antimicrobial potency	60
*Clinacanthus nutans*	Leaves	Stir and reflux, (60–100) °C for 1–6 h	UV-vis peak: 270 nm, 2*θ*: 22.12°, *I*_D_/*I*_G_ (rGO): 1.08 > *I*_D_/*I*_G_ (GO): 1.01	n.d.	61
*Acorus calamus*	Rhizome	Sonication, 1–2 h	UV-vis peak: 278 nm, 2*θ*: 26.4°, dense, compact structure	Antibacterial efficacy	62
*Terminalia bellirica*	Fruit and seed	Sonication, 1–2 h	UV-vis peak: 262 nm, 2*θ*: 26.4°, layered structure	Antibacterial efficacy	62
*Helicteres isora*	Fruit and seed	Sonication, 1–2 h	UV-vis peak: 268 nm, 2*θ*: 26.4°, layered structure	Antibacterial efficacy	62
*Quercus infectoria*	Fruit and seed	Sonication, 1–2 h	UV-vis peak: 263 nm, 2*θ*: 26.4°, staked, crumpled and flaky	Antibacterial efficacy	62
*Turbinella pyrum*	Shell	Sonication, 1–2 h	UV-vis peak: 264 nm, 2*θ*: 26.4°, layered structure	Antibacterial efficacy	62
*Vitis vinifera*	Fruit	Reflux, 95 °C for 1–6 h	UV-vis peak: 270 nm, 2*θ*: 23.7°	Removal of dye	63
*Murraya koenigii*	Leaves	Autoclave, 100 °C for 12 h	UV-vis peak: 270 nm, 2*θ*: 26.25°, *I*_D_/*I*_G_ (rGO): 1.14 > *I*_D_/*I*_G_ (GO): 1.02	Photocatalysis	64
*Catharanthus roseus*	Roots	Stir, 24 h	*I*_D_/*I*_G_ (rGO): 1.20 > *I*_D_/*I*_G_ (GO): 0.93	n.d.	65
*Phyllarthron madagascariense* K. Schum	Leaves	Stir, 24 h	*I*_D_/*I*_G_ (rGO): 1.17 > *I*_D_/*I*_G_ (GO): 1.01	n.d.	65
*Cinnamomum camphora* cineoliferum	Leaves	Stir, 24 h	*I*_D_/*I*_G_ (rGO): 1.21 > *I*_D_/*I*_G_ (GO): 1.01	n.d.	65
*Cedrelopsis grevei* Baill	Barks	Stir, 24 h	*I*_D_/*I*_G_ (rGO): 1.26 > *I*_D_/*I*_G_ (GO): 0.93	n.d.	65
Lemon juice	Fruit	Stir, 80 °C for 2 h	UV-vis peak: 272 nm, 2*θ*: 24.26°, *I*_D_/*I*_G_ (rGO): 1.05 > *I*_D_/*I*_G_ (GO): 1.04	Adsorption of methylene blue	66
Mango	Leaves	Stir and reflux, 90 °C for 24 h	UV-vis peak: 272 nm, 2*θ* = 25.53°, C/O ratio: 3.75	n.d.	50
Potato	Vegetable	Stir and reflux, 90 °C for 24 h	UV-vis peak: 277 nm, 2*θ* = 21.34°, C/O ratio: 3.77	n.d.	50
Banana	Peel	Stir and reflux, 90 °C for 24 h	UV-vis peak: 280 nm, 2*θ* = 22.89°, C/O ratio: 3.80	n.d.	50
Rose water	Flower	Stir, RT for (70–100) °C	2*θ* = 24°, C/O ratio: 2.97	n.d.	67
Wild carrot	Roots	Stir, RT for 48 h	2*θ* = 23.96°, C/O ratio: 11.9, *I*_D_/*I*_G_ (rGO): 1.06 > *I*_D_/*I*_G_ (GO): 0.80	n.d.	48
Palm	Leaves	Reflux, 100 °C for 3 h	2*θ* = 24.5°	n.d.	68
*Hibiscus sabdariffa* L.	Flower	Stir, RT for 1 h	UV-vis peak: 262.8 nm, 2*θ* = 25.0°, *I*_D_/*I*_G_ (rGO): 1.24 > *I*_D_/*I*_G_ (GO): 1.01	Supercapacitor	69
*Ficus carica*	Leaves	Reflux, 98 °C for 1–30 h	UV-vis peak: 270 nm, 2*θ* = 24.50° and 43°	n.d.	70
*Phragmites australis*	Leaves	Reflux, 98 °C for 1–30 h	UV-vis peak: 267 nm, 2*θ* = 24.50° and 43°	n.d.	70
Sweet potato	Vegetable	Reflux, 80 °C for 3 h	UV-vis peak: 269 nm, *I*_D_/*I*_G_ (rGO): 0.97 > *I*_D_/*I*_G_ (GO): 0.94	n.d.	71
*Bougainvillea glabra*	Flower	Stir, 95 °C for 5 h	UV-vis peak: 270 nm, C/O ratio: 4.6	Sensing	72
*Citrus grandis*	Fruit	Reflux, 95 °C for 12 h	UV-vis peak: 270 nm, 2*θ* = 24.5°, *I*_D_/*I*_G_ (rGO): 1.14 > *I*_D_/*I*_G_ (GO): 0.86	Supercapacitor	73
*Tamarindus indica*	Fruit	Reflux, 95 °C for 12 h	UV-vis peak: 275 nm, 2*θ* = 24.9°, *I*_D_/*I*_G_ (rGO): 1.16 > *I*_D_/*I*_G_ (GO): 0.86	Supercapacitor	73
*Chrysanthemum*	Flower	Water bath, 95 °C for 24 h	2*θ* = 24.6°, C/O ratio: 4.96, *I*_D_/*I*_G_ (rGO): 1.14 > *I*_D_/*I*_G_ (GO): 0.896	n.d.	41
*Lycium barbarum*	Fruit	Water bath, 95 °C for 24 h	2*θ* = 26°, C/O ratio: 4.96, *I*_D_/*I*_G_ (rGO): 1.05 > *I*_D_/*I*_G_ (GO): 0.896	n.d.	74
Tea	Leaves	Stir, 80 °C for 1 h	C/O ratio: 3.88, *I*_D_/*I*_G_ (rGO): 1.02 > *I*_D_/*I*_G_ (GO): 1.005	n.d.	75
*Syzygium samarangense*	Fruit	Stir, 60 °C for 40 h	2*θ* = 23.78°, *I*_D_/*I*_G_ (rGO): 1.17 > *I*_D_/*I*_G_ (GO): 0.92	n.d.	76
Sugarcane bagasse	Agro waste	Stir, 95 °C for 12 h	UV-vis peak: 270 nm, C/O ratio: 4.27, *I*_D_/*I*_G_ (rGO): 1.16 > *I*_D_/*I*_G_ (GO): 0.98	Removal of cadmium	77
*Larrea tridentata*	Flower	Reflux, 80 °C for 12 h	UV-vis peak: 280 nm, *I*_D_/*I*_G_ (rGO): 0.983 < *I*_D_/*I*_G_ (GO): 0.99	Photocatalysis	78
*Capsicum chinense*	Vegetable	Reflux, 80 °C for 12 h	UV-vis peak: 260 nm, *I*_D_/*I*_G_ (rGO): 0.987 < *I*_D_/*I*_G_ (GO): 0.99	Photocatalysis	78
*Ocimum sanctum* L.	Leaves	Stir, 70 °C for 4 h	UV-vis peak: 267.8 nm, C/O ratio: 3.10	n.d.	79
*Acalypha indica*	Leaves	Autoclave, 100 °C for 12 h	UV-vis peak: 272 nm, *I*_D_/*I*_G_ (rGO): 1.22 > *I*_D_/*I*_G_ (GO): 1.02	Cytotoxicity	80
*Raphanus sativus*	Root	Autoclave, 100 °C for 12 h	UV-vis peak: 282 nm, *I*_D_/*I*_G_ (rGO): 1.15 > *I*_D_/*I*_G_ (GO): 1.02	Cytotoxicity	80
*Aloe vera*	Leaves	Stir, 95 °C for 24 h	UV-vis peak: 259 nm	Electrochemical analysis and dye removal	81
*Salvadora persica* L.	Root	Reflux, 98 °C for 24 h	UV-vis peak: 280 nm, 2*θ* = 22.4°	n.d.	82
*Citrus hystrix*	Peel	Stir, RT for 8 h	UV-vis peak: 300 nm, 2*θ* = 8.75° and 26.34°	Methylene blue adsorption	83
*Tecoma stans*	Leaves	Stir, 70 °C for 12 h	UV-vis peak: 280 nm	Removal of Ni(ii)	84
*Salvia spinosa*	Leaves	Reflux, 95 °C for 12 h	UV-vis peak: 274 nm, 2*θ* = 26.2°, *I*_D_/*I*_G_ (rGO): 0.91 < *I*_D_/*I*_G_ (GO): 0.95	Evaluation of photothermal effect	85
*Mangifera indica*	Leaves	Reflux, 70–80 °C for 12 h	UV-vis peak: 259 nm, 2*θ* = 21.87°, *I*_D_/*I*_G_ (rGO): 1.024 > *I*_D_/*I*_G_ (GO): 0.846	Electrical conductivity analysis	29
*Solanum tuberosum* L.	Vegetable	Reflux, 70–80 °C for 12 h	UV-vis peak: 265 nm, 2*θ* = 21.86°, *I*_D_/*I*_G_ (rGO): 1.066 > *I*_D_/*I*_G_ (GO): 0.846	Electrical conductivity analysis	29
*Tinospora cordifolia*	Stem	Reflux, 85 °C for 3 h	UV-vis peak: 263 nm, 2*θ* = 22.81°	Dye degradation and antibacterial activity	86
*Ocimum sanctum*	Leaves	Reflux, 100 °C for 10 h	2*θ* = 25°	Cytotoxicity	87
Spinach	Leaves	Reflux, RT for 30 min	UV-vis peak: 282 nm, 2*θ* = 26°	Antioxidant and dye adsorption	88
*Citrus hystrix*	Peel	Stir, RT for 8 h	C–C/C–O ratio: 1.07, 2*θ* = 10.15°	Methylene blue adsorption	89
*Punica granatum* L.	Seed	Reflux, 98 °C for 8 h	UV-vis peak: 280 nm	Antioxidant	90
*Prunus serrulata*	Leaves	Reflux, 95 °C for 12 h	UV-vis peak: 272 nm, C/O ratio: 5.10, 2*θ* = 26.2°	n.d.	91
*Magnolia kobus*	Leaves	Reflux, 95 °C for 12 h	C/O ratio: 4.40	n.d.	91
*Platanus orientalis*	Leaves	Reflux, 95 °C for 12 h	C/O ratio: 4.96	n.d.	91
*Eclipta prostrata*	Leaves	Stir, RT for 4 h	C/O ratio: 2.70	n.d.	92
*Eichhornia crassipes*	Whole except the root	Reflux	UV-vis peak: 274 nm, 2*θ* = 26°	n.d.	93
*Pulicaria glutinosa*	Whole plant	Stir, 98 °C for 24 h	UV-vis peak: 280 nm, 2*θ* = 22.4°	n.d.	94
*Rhus coriaria*	Fruit	Reflux, 95 °C for 12 h	UV-vis peak: 282 nm, 2*θ* = 26.91°, *I*_D_/*I*_G_ (rGO): 1.04 > *I*_D_/*I*_G_ (GO): 0.84	Cytotoxicity	95
Olive	Leaves	Reflux, 100 °C for 10 h	UV-vis peak: 270 nm, 2*θ* = 24.6°	n.d.	46
*Annona squamosa*	Leaves	Reflux, 100 °C for 12 h	UV-vis peak: 276 nm, 2*θ* = 23°	n.d.	96
Green coffee bean	Fruit	Stir, 80 °C for 12 h	UV-vis peak: 275 nm, 2*θ* = 22°, *I*_D_/*I*_G_ (rGO): 1.02 < *I*_D_/*I*_G_ (GO): 1.04	Dye removal	97
*Mangifera indica*	Leaves	Stir, 50 °C for 24 h	UV-vis peak: 266 nm, *I*_D_/*I*_G_ (rGO): 1.21 > *I*_D_/*I*_G_ (GO): 1.10	Highly conductive film	98
*Ficus religiosa*	Leaves	Stir, 50 °C for 24 h	*I*_D_/*I*_G_ (rGO): 1.12 > *I*_D_/*I*_G_ (GO): 1.10	Highly conductive film	98
*Polyalthia longifolia*	Leaves	Stir, 50 °C for 24 h	*I*_D_/*I*_G_ (rGO): 1.18 > *I*_D_/*I*_G_ (GO): 1.10	Highly conductive film	98
Ginger	Root	Stir, 90 °C for 24 h	2*θ* = 24.34°, *I*_D_/*I*_G_ (rGO): 0.91 < *I*_D_/*I*_G_ (GO): 1.14	Supercapacitor	99
*Urtica dioica*	Leaves	Ultra-sonication, 90 °C for 1 h	UV-vis peak: 259 nm, *I*_D_/*I*_G_ (rGO): 1.13 > *I*_D_/*I*_G_ (GO): 0.91	Antioxidant	42
*Colocasia esculenta*	Leaves	Reflux, 5 h	UV-vis peak: 270.9 nm	n.d.	44
*Mesua ferrea* Linn.	Leaves	Reflux, 8 h	UV-vis peak: 268 nm	n.d.	44
*Terminalia chebula*	Seed	Reflux in a water bath, 90 °C for 24 h	UV-vis peak: 275 nm, 2*θ* = 26.6°, *I*_D_/*I*_G_ (rGO) > *I*_D_/*I*_G_ (GO)	n.d.	52
Eucalyptus	Bark	Reflux, 80–85 °C for 24 h	UV-vis peak: 270 nm, 2*θ* = 25°, *I*_D_/*I*_G_ (rGO): 1.15 > *I*_D_/*I*_G_ (GO): 0.98	Supercapacitor	100
Tulsi (holy basil) green tea	Leaves	Microwave irradiation at 800 W for 1 min	UV-vis peak: 270 nm, 2*θ* = 26.35°, *I*_D_/*I*_G_ (rGO): 1.40 > *I*_D_/*I*_G_ (GO): 1.08	Supercapacitor and dye removal	101

a n.d. = not done.

#### 3.1.2 Proposed reduction mechanisms

**Fig. 4:**
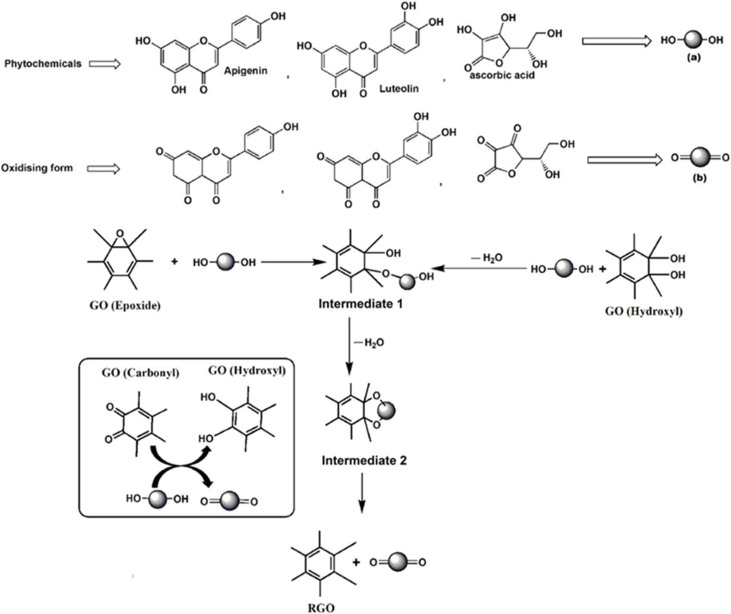
Proposed reaction mechanism for the chemical reduction of GO by polyphenols (*e.g.*, luteolin, apigenin), proceeding *via* a nucleophilic attack on epoxide and hydroxyl groups followed by dehydration. Reproduced from ref. 44 (Thakur and Karak) with permission from Elsevier, copyright 2012.

**Fig. 5:**
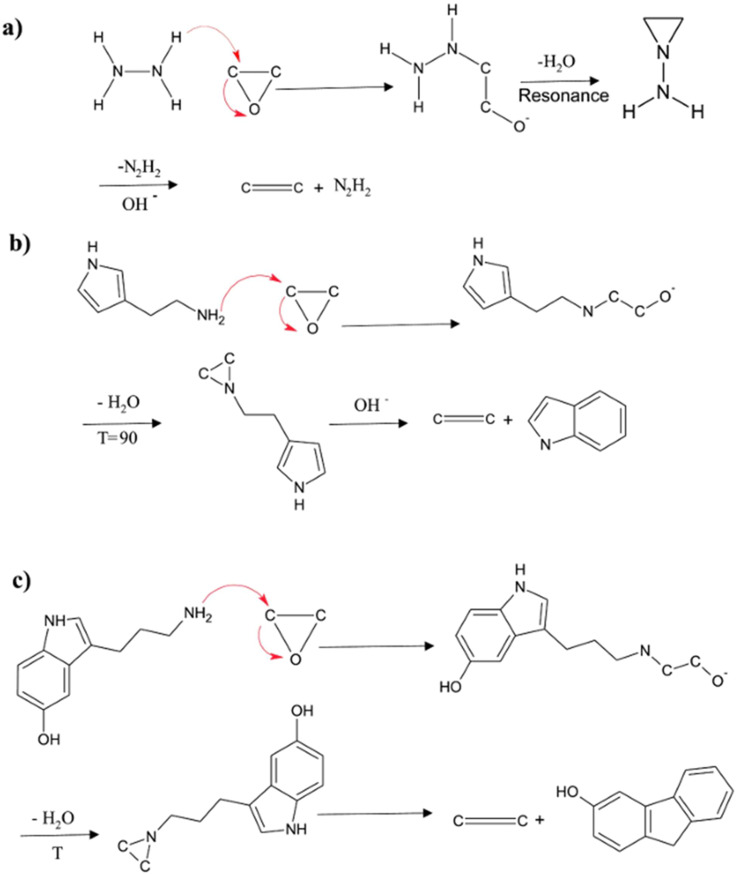
Comparison of the proposed reduction mechanism for epoxide groups on GO by (a) hydrazine, (b) histamine, and (c) serotonin, all involving a nucleophilic attack by the amine group. Reproduced from ref. 42 (Mahmudzadeh, *et al.*) with permission from Elsevier, copyright 2019.

The most widely accepted mechanism for GO reduction by polyphenol-rich plant extracts involves a nucleophilic attack. As shown in Fig. 4, the hydroxyl groups of polyphenols (like luteolin) are deprotonated, and the resulting oxygen anion acts as a nucleophile, attacking the electrophilic carbon of an epoxide group on the GO basal plane. It leads to a ring-opening reaction. Subsequent dehydration restores the sp^2^ C=C bond, forming rGO, while the polyphenol is oxidized to a quinone-type structure.^44^ However, other mechanisms are also proposed. For artemisinin, a free-radical-driven mechanism is proposed, in which heating the endoperoxide bridge generates hydroxyl radicals that aggressively attack and remove all types of oxygen functional groups.^47^ This radical pathway may explain the superior deoxygenation (C/O ratio of 11.7) compared to the more selective nucleophilic pathway of polyphenols. In another case, the amine groups in histamine and serotonin from nettle extract were proposed to reduce GO *via* a mechanism similar to hydrazine, involving a nucleophilic attack followed by elimination (Fig. 5).^42^ The diversity of these mechanisms highlights that the “plant extract” category is not monolithic; the specific chemistry of the dominant phytochemical dictates the reduction pathway and, ultimately, the quality of the final rGO.

#### 3.1.3 Application-specific properties and outlook

The properties of plant-extract-synthesized rGO make it suitable for a range of applications, particularly in environmental remediation and energy storage.

##### 3.1.3.1 Environmental remediation

The reduction process with plant extracts is often incomplete, leaving residual oxygen functional groups on the rGO surface. While detrimental for conductivity, these groups act as excellent binding sites for adsorbing heavy metal ions and organic dyes. For instance, rGO produced with *Citrus hystrix* peel extract showed an impressive dye-removal efficacy with a maximum adsorption capacity (*q*_max_) of 276.06 mg g^−1^ at room temperature,^83^ while tulsi green tea extract-derived rGO could remove MG with a maximum adsorption capacity of 416.7 mg g^−1^.^101^ On the other hand, Mahmoud, *et al.*^84^ utilized *Tecoma stans* extracts to synthesize rGO for removing Ni(ii) with a maximum uptake capacity (*q*_max_) of 69 mg g^−1^. The combination of a restored π-system (for π–π stacking interactions with aromatic dyes) and residual polar groups (for electrostatic and hydrogen bonding interactions) makes this type of rGO a highly effective adsorbent.

##### 3.1.3.2 Energy storage

For supercapacitor applications, a high specific surface area is paramount for enabling ion adsorption at the electrode–electrolyte interface. The use of certain plant extracts, such as lemon juice, yields rGO with a moderate degree of reduction but a high specific surface area (159 m^2^ g^−1^). Such property is particularly advantageous for supercapacitors, as demonstrated by the specific capacitance of 124 F g^−1^ reported by Joshi, *et al.*^17^ Moreover, an excellent specific capacitance of 239 F g^−1^ was observed for the rGO synthesized using eucalyptus bark extract.^100^ This highlights how the morphological properties imparted by certain green reductants can be tailored for specific energy storage needs.

### 3.2 Reduction of GO using microorganisms

**Fig. 6:**
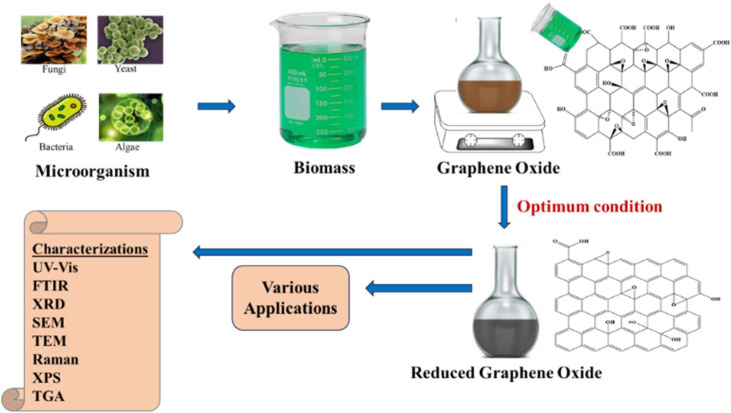
Schematic illustration of the general process for microbial reduction of GO.

Microorganisms, including bacteria, fungi, and yeast, offer a sustainable and biocompatible route for rGO synthesis. These methods leverage the metabolic and enzymatic machinery of microbes to reduce GO under mild conditions, often at room temperature or slightly above (Fig. 6).

#### 3.2.1 General methodology and reduction efficacy

In a typical microbial reduction, a GO dispersion is introduced into a microbial culture (*e.g.*, bacteria in a nutrient broth) and incubated for a period ranging from hours to several days. The microbial cells or their secreted enzymes interact with the GO sheets, facilitating electron transfer and reduction. The bacterial species *Shewanella* has been extensively studied for this purpose, as it can reduce GO under both anaerobic and aerobic conditions.^31,102,103^ The reduction efficacy is generally good, with C/O ratios reaching up to 5.9 for yeast-mediated reduction^104^ and electrical conductivity of 55.32 S m^−1^ for *Shewanella*-reduced GO.^31^ The main drawback of microbial methods is the long reaction time (often 24–168 h) and the need for sterile culture conditions, which can complicate scalability. A summary of representative studies is provided in Table 2.

**Table 2:** Summary of microorganism-mediated reduction of GO, with key characterization data^a^

Species	Reduction conditions	Characteristics	Applications	Ref.
**Bacteria**				
*Lactococcus lactis*	Aerobic, 30 °C for 7 days	2*θ* = 26.5°, C/O ratio: 3.70, *I*_D_/*I*_G_ (rGO): 1.35 < *I*_D_/*I*_G_ (GO): 2.41	Cytotoxicity	105
*Lactobacillus plantarum*	Aerobic, 30 °C for 7 days	2*θ* = 26.5°, C/O ratio: 2.98, *I*_D_/*I*_G_ (rGO): 1.46 < *I*_D_/*I*_G_ (GO): 2.41	Cytotoxicity	105
*Escherichia coli*	Aerobic, 37 °C for 7 days	2*θ* = 26.5°, C/O ratio: 2.80, *I*_D_/*I*_G_ (rGO): 1.26 < *I*_D_/*I*_G_ (GO): 2.41	Cytotoxicity	105
*Shewanella oneidensis*	Aerobic and anaerobic, RT for 2 days	*I*_D_/*I*_G_ (rGO): 1.00 ± 0.09 > *I*_D_/*I*_G_ (GO): 0.85 ± 0.03	Creation of conductive graphene materials	31
*Shewanella oneidensis* MR-1	Aerobic and anaerobic, RT for different time intervals	C/O ratio: increasing over time	n.d.	103
*Enterobacter cloacae*	Aerobic, 20–25 °C for 3 days	UV-vis peak: 270 nm, *I*_D_/*I*_G_ (rGO): 1.17 > *I*_D_/*I*_G_ (GO): 1.09	n.d.	106
*Bacillus* sp.	Aerobic, 20–25 °C for 3 days	UV-vis peak: 270 nm, *I*_D_/*I*_G_ (rGO): 1.20 > *I*_D_/*I*_G_ (GO): 1.09	n.d.	106
*Shewanella baltica*	Aerobic, 20–25 °C for 3 days	UV-vis peak: 270 nm, *I*_D_/*I*_G_ (rGO): 0.99 < *I*_D_/*I*_G_ (GO): 1.09	n.d.	106
*Shewanella oneidensis* MR-1	Anaerobic, RT for 3 days	% C–C: 56%	n.d.	102
*Shewanella putrefaciens* CN32	Anaerobic, RT for 3 days	% C–C: 91%	n.d.	102
*Shewanella amazonensis* SB2B	Anaerobic, RT for 3 days	% C–C: 75%	n.d.	102
*Shewanella putrefaciens* W3-18-1	Anaerobic, RT for 3 days	% C–C > 95%	n.d.	102
*Shewanella baltica* 10735	Anaerobic, RT for 3 days	% C–C: 54%	n.d.	102
*Escherichia coli*	37 °C for 3 days	UV-vis peak: 267 nm, 2*θ* = 24°	n.d.	22
*Bacillus sphaericus*	30 °C for 2 days	UV-vis peak: 261 nm, C/O ratio: 2.62, *I*_D_/*I*_G_ (rGO): 1.17 > *I*_D_/*I*_G_ (GO): 0.99	n.d.	107
*Azotobacter chroococcum*	RT for 72 h	C/O ratio: 4.18, 2*θ* = 17–24°	n.d.	108
*Desulfovibrio desulfuricans*	25 °C for 24 h	2*θ* = 17–24°, *I*_D_/*I*_G_ (rGO): 1.13 > *I*_D_/*I*_G_ (GO): 0.92	Anti-biocorrosion	109
*Escherichia coli*	Aerobic, 37 °C for 0.5 h	2*θ* = 26.6°, C/O ratio: 5.78, *I*_D_/*I*_G_ (rGO): 0.72 < *I*_D_/*I*_G_ (GO): 0.95	Superoxide formation	110
*Shewanella* sp. CF8-6	Facultative anaerobic, 25 °C for 12 h	2*θ* = 23.1°, *I*_D_/*I*_G_ (rGO): 1.26 > *I*_D_/*I*_G_ (GO): 1.11	Dye adsorption	39
*Shigella dysenteriae*	37 °C for 10 h	2*θ* = 20–23°, *I*_D_/*I*_G_ (rGO): 1.15 > *I*_D_/*I*_G_ (GO): 0.84	n.d.	111
*G. sulfurreducens*	30 °C for 9 days	O/C ratio: 0.49, *I*_D_/*I*_G_ (rGO): 1.324 > *I*_D_/*I*_G_ (GO): 0.945	n.d.	112
*Bacillus subtilis* 168	25 ± 2 °C for time intervals	2*θ* = 24.18°, *I*_D_/*I*_G_ (rGO): 1.01 > *I*_D_/*I*_G_ (GO): 0.92	n.d.	113
*Shewanella decolorationis* NTOU1	35 °C for 24 h	C/O ratio: 3.0	n.d.	114
*Escherichia coli* strain E-NO.7	37 °C for 72 h	2*θ* = 26.5°	n.d.	115
*Lactobacillus plantarum*	30 °C for 7 days	C/O ratio: 3.3, *I*_D_/*I*_G_ (rGO): 0.92 < *I*_D_/*I*_G_ (GO): 0.94	n.d.	116
*Lactococcus lactis*	30 °C for 3–4 days	2*θ* = 23°–26°, *I*_D_/*I*_G_ (rGO): 0.97 < *I*_D_/*I*_G_ (GO): 2.15	n.d.	117
*Bacillus clausii*	37 °C for 72 h	UV-vis peak: 268 nm, 2*θ* = 24.5°	Against MDR uropathogenic isolates	118
*Pseudoalteromonas* sp. CF10-13	Facultative anaerobic, 25 °C for 12 h	2*θ* = 21.9°, *I*_D_/*I*_G_ (rGO): 1.3 > *I*_D_/*I*_G_ (GO): 1.03	n.d.	119
*Gluconobacter roseus*	37 °C for 24 h	UV-vis peak: 280 nm, *I*_D_/*I*_G_ (rGO): 0.87 < *I*_D_/*I*_G_ (GO): 1.12	Electrochemical study	120
*Escherichia fergusoni*	37 °C for 72 h	UV-vis peak: 267 nm, 2*θ* = 25.6°, *I*_D_/*I*_G_ (rGO): 1.96 > *I*_D_/*I*_G_ (GO): 1.58	n.d.	121

**Fungi**				
*Rhizopus oryzae*	Shaking, 37 °C for 24 h	2*θ* = 26.07°, *I*_D_/*I*_G_ (rGO): 1.17 > *I*_D_/*I*_G_ (GO): 0.96	Antimicrobial coating for medical devices	23
*Ganoderma* spp.	Ultrasonicated, 40 °C for 24 h	UV-vis peak: 265 nm, 2*θ* = 26.5°, *I*_D_/*I*_G_ (rGO): 2.1 > *I*_D_/*I*_G_ (GO): 1.8	Cytotoxicity	122
*Aspergillus* sp.	Static, 40 °C for 72 h	UV-vis peak: 270 nm, *I*_D_/*I*_G_ (rGO): 1.06 > *I*_D_/*I*_G_ (GO): 1.01	Antibacterial study	123
*Ganoderma lucidum*	Water bath, 85 °C for 16 h	UV-vis peak: 260 nm, 2*θ* = 24.0°, *I*_D_/*I*_G_ (rGO): 0.99 > *I*_D_/*I*_G_ (GO): 0.94	n.d.	124

**Algae**				
*Chlorella* sp.	Water bath, 90 °C for 96 h	UV-vis peak: 267 nm, *I*_D_/*I*_G_ (rGO): 0.935 > *I*_D_/*I*_G_ (GO): 0.853	Biophotovoltaic devices	24
*Turbinaria ornata*	Water bath, 60 °C for NA	UV-vis peak: 267 nm, 2*θ* = 26.4°	Cytotoxicity	125

**Yeast**				
Baker's yeast	Stir, 35–40 °C for 72 h	UV-vis peak: 264 nm, 2*θ* = 23.5°, C/O ratio: 5.9, *I*_D_/*I*_G_ (rGO): 1.44 > *I*_D_/*I*_G_ (GO): 0.8	n.d.	104

a n.d. = not done.

#### 3.2.2 Proposed reduction mechanisms

Microbial reduction of GO can occur through several distinct pathways, primarily involving electron transfer from the cell's respiratory chain.

##### 3.2.2.1 Direct electron transfer

**Fig. 7:**
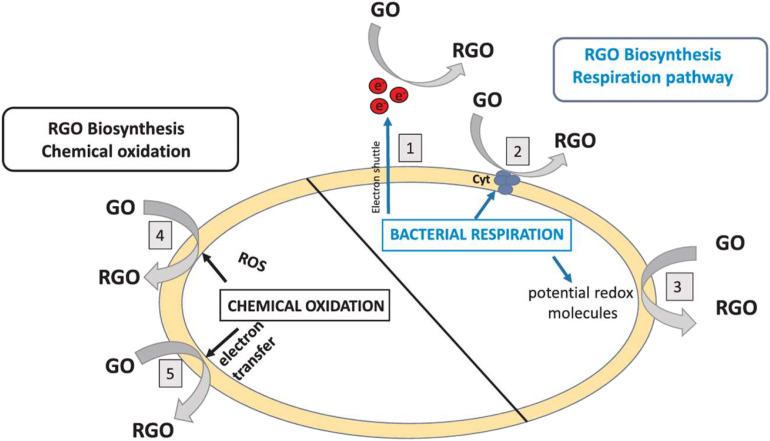
Proposed bacterial reduction strategies for GO, mediated by either bacterial respiration (paths 1–3) or chemical oxidation *via* lysis and release of intracellular components (paths 4 and 5). Reproduced from ref. 106 (Vargas, *et al.*) with permission from Elsevier, copyright 2019.

Electrochemically active bacteria like *Shewanella* possess outer-membrane cytochromes (*e.g.*, the Mtr pathway) that can directly transfer electrons to GO when it acts as a terminal electron acceptor, analogous to how they reduce metal oxides (Fig. 7, path 2).^102,126^

##### 3.2.2.2 Indirect electron transfer *via* mediators

Some bacteria secrete redox-active molecules (electron shuttles) like flavins, which can accept electrons from the cell and subsequently shuttle them to external GO sheets (Fig. 7, path 1).^31^

##### 3.2.2.3 Enzymatic reduction

Specific enzymes, such as nitrogenase from *Azotobacter chroococcum*, have been proposed to directly catalyze the reduction of GO through a series of proton and electron additions followed by dehydration.^108^

##### 3.2.2.4 Chemical oxidation of cellular components

An alternative pathway suggests that the antibacterial properties of GO itself can induce oxidative stress and membrane damage in microbes. It leads to the leakage of intracellular reducing components (*e.g.*, NADH, glutathione) that then chemically reduce the GO (Fig. 7, paths 4 and 5).^106^

#### 3.2.3 Application-specific properties and outlook

The inherent biocompatibility of microbially synthesized rGO makes it exceptionally well-suited for biomedical applications.

##### 3.2.3.1 Biomedical applications

Because the reduction occurs in a biological environment and often involves biomolecules that remain on the rGO surface, the resulting material exhibits low cytotoxicity. Utkan, *et al.*^105^ demonstrated that rGO produced by *Lactococcus lactis* and *Lactobacillus plantarum* exhibited minimal cytotoxicity against human cell lines, making it a safe candidate for biomedical use. Furthermore, the rGO itself often exhibits potent antibacterial activity. Akhavan and Ghaderi^127^ showed that *E. coli* reduces GO to a bactericidal form of graphene, likely due to the sharp edges of the restored nanosheets, which induce membrane stress. This dual function—biocompatible synthesis leading to an antimicrobial product—is a unique advantage of this method.

##### 3.2.3.2 Anti-biocorrosion coatings

rGO's impermeability and hydrophobicity make it an excellent barrier against corrosive agents. Song, *et al.*^109^ demonstrated that *D. desulfuricans*, a bacterium responsible for biocorrosion, could be used to reduce a GO coating on a copper surface. The resulting *in situ*-formed rGO layer then acted as a protective barrier, inhibiting further corrosion by the same bacteria.

### 3.3 Reduction of GO using biomolecules

Using well-defined, pure biomolecules as reducing agents offers a “best of both worlds” approach, combining the eco-friendliness of natural compounds with the precision and control of synthetic chemistry. This strategy allows for better reproducibility and the ability to tune the properties of the final rGO product.

#### 3.3.1 General methodology and reduction efficacy

This method is experimentally straightforward, involving the addition of a pure biomolecule solution (*e.g.*, ascorbic acid, l-cysteine, glucose) to a GO dispersion, typically with mild heating. Ascorbic acid (vitamin C) is one of the most effective and widely studied green reductants. A seminal study by Fernández-Merino, *et al.*^128^ showed that ascorbic acid could achieve a degree of reduction comparable to hydrazine, yielding an excellent O/C ratio of 0.08 (equivalent to a C/O ratio of 12.5). The results demonstrated that green reductants could match the performance of hazardous chemicals. Other biomolecules like gallic acid (C/O ratio: 3.89)^30^ and caffeic acid (C/O ratio: 7.15)^40^ are also highly effective. The use of pure compounds provides excellent control over the reaction, but the cost of purified biomolecules can be a limiting factor for large-scale production compared to crude plant extracts. A summary of various biomolecules used as reductants is presented in Table 3.

**Table 3:** Summary of biomolecule-mediated reduction of GO, with key characterization data^a^

Biomolecules	Reduction conditions	Characteristics	Applications	Ref.
Glucose	Ultrasonic bath, RT for 60 min	UV-vis peak: 270 nm, 2*θ*: 20.78°, C/O ratio: 2.2	n.d.	129
Ascorbic acid and sodium citrate binary mixture	Ultrasonic bath, RT for 24 h	UV-vis peak: 260 nm, 2*θ*: 23.54°	n.d.	130
β-Carotene	Reflux, 95 °C for 24 h	UV-vis peak: 270 nm, 2*θ*: 23.13°, *I*_D_/*I*_G_ (rGO): 1.01 > *I*_D_/*I*_G_ (GO): 0.86	Supercapacitor	131
Gallic acid	Stir, RT for 24 h	UV-vis peak: 270 nm, 2*θ*: 26°, C/O ratio: 3.89, *I*_D_/*I*_G_ (rGO): 1.92 > *I*_D_/*I*_G_ (GO): 1.74	n.d.	30
Tannic acid	Sonication, 80 °C for 10 h	UV-vis peak: 274 nm, C/O ratio: 1.21, *I*_D_/*I*_G_ (rGO): 1.18 > *I*_D_/*I*_G_ (GO): 0.97	n.d.	132
Honeycomb flavone chrysin	Stir, 90 °C for 1 h	2*θ*: 24.6°, *I*_D_/*I*_G_ (rGO): 1.755 > *I*_D_/*I*_G_ (GO): 1.518	Improved bactericidal and skin regeneration	133
Starch	Reflux, 80 °C for 3 h	UV-vis peak: 269 nm, 2*θ*: 21.3°, *I*_D_/*I*_G_ (rGO): 0.97 > *I*_D_/*I*_G_ (GO): 0.94	n.d.	71
Ascorbic acid	Stir, 95 °C for 1 h	2*θ*: 23.8°	n.d.	134
Ascorbic acid	Stir, 60 °C for 12 h	UV-vis peak: 308 nm, 2*θ*: 24.10°	n.d.	135
Ascorbic acid	Spray, 50 °C for 48 h	2*θ*: 25.39°	n.d.	136
Ascorbic acid	95 °C for 30 min	UV-vis peak: 266 nm, O/C ratio: 0.08	n.d.	128
Dopamine	Stir, 60 °C for 2 h	2*θ*: 21.88°, *I*_D_/*I*_G_ (rGO): 1.06 > *I*_D_/*I*_G_ (GO): 0.87	Flexible film	33
Uric acid	Incubate, 40 °C for 1 h and stir, 90 °C for 1 h	UV-vis peak: 260 nm, 2*θ*: 25.9°, *I*_D_/*I*_G_ (rGO): 2.02 > *I*_D_/*I*_G_ (GO): 1.5	Anticancer agent	137
Citric acid	Ultrasonic bath, 92 °C for 1.5 h	UV-vis peak: 268 nm, *I*_D_/*I*_G_ (rGO): 1.29 > *I*_D_/*I*_G_ (GO): 1.09	Adsorption of dye	138
Ethanol	Reflux, 150 °C for 8 h	2*θ*: 24.40°, C/O ratio: 2.72	Superconductor	139
Caffeic acid	Stir, 95 °C for 24 h	2*θ*: 24.89°, C/O ratio: 7.15, *I*_D_/*I*_G_ (rGO): 1.15 > *I*_D_/*I*_G_ (GO): 0.86	Sensing and energy storage	40
Alanine	Stir, 85 °C for 24 h	UV-vis peak: 258 nm, *I*_D_/*I*_G_ (rGO): 0.996 > *I*_D_/*I*_G_ (GO): 0.943	n.d.	140
Extracellular polymeric substances	Stir, 40 °C for 24 h	C/O ratio: 3.18, *I*_D_/*I*_G_ (rGO): 1.0183 < *I*_D_/*I*_G_ (GO): 1.0375	n.d.	141
Enhanced green fluorescent protein	Ultrasonication, 40 °C for 15 min and water bath, 90 °C for 1 h	UV-vis peak: 258 nm, 2*θ*: 25.8°, *I*_D_/*I*_G_ (rGO): 2.149 > *I*_D_/*I*_G_ (GO)	n.d.	142
Nicotinamide	Stir, 40 °C for 6 h	UV-vis peak: 260 nm, 2*θ*: 26.2°, *I*_D_/*I*_G_ (rGO): 1.74 > *I*_D_/*I*_G_ (GO): 1.01	Cytotoxicity	143
l-Glutathione	Ultrasonication for 1 h and 50 °C for 6 h	2*θ*: 24.7°	n.d.	144
Lignin	Autoclave, 180 °C for 12 h	UV-vis peak: 270 nm, O/C ratio: 0.286	Electrochemical property	35
Melatonin	Ultrasonication, 80 °C for 3 h	UV-vis peak: 269 nm, *I*_D_/*I*_G_ (rGO): 1.07 < *I*_D_/*I*_G_ (GO): 1.21	Antioxidant	145
Humanin	Ultrasonication for 15 min and 40 °C for 1 h	UV-vis peak: 265 nm, 2*θ*: 26.4°, *I*_D_/*I*_G_ (rGO): 2.3 > *I*_D_/*I*_G_ (GO): 1.4	n.d.	146

a n.d. = not done.

#### 3.3.2 Proposed reduction mechanisms

**Fig. 8:**
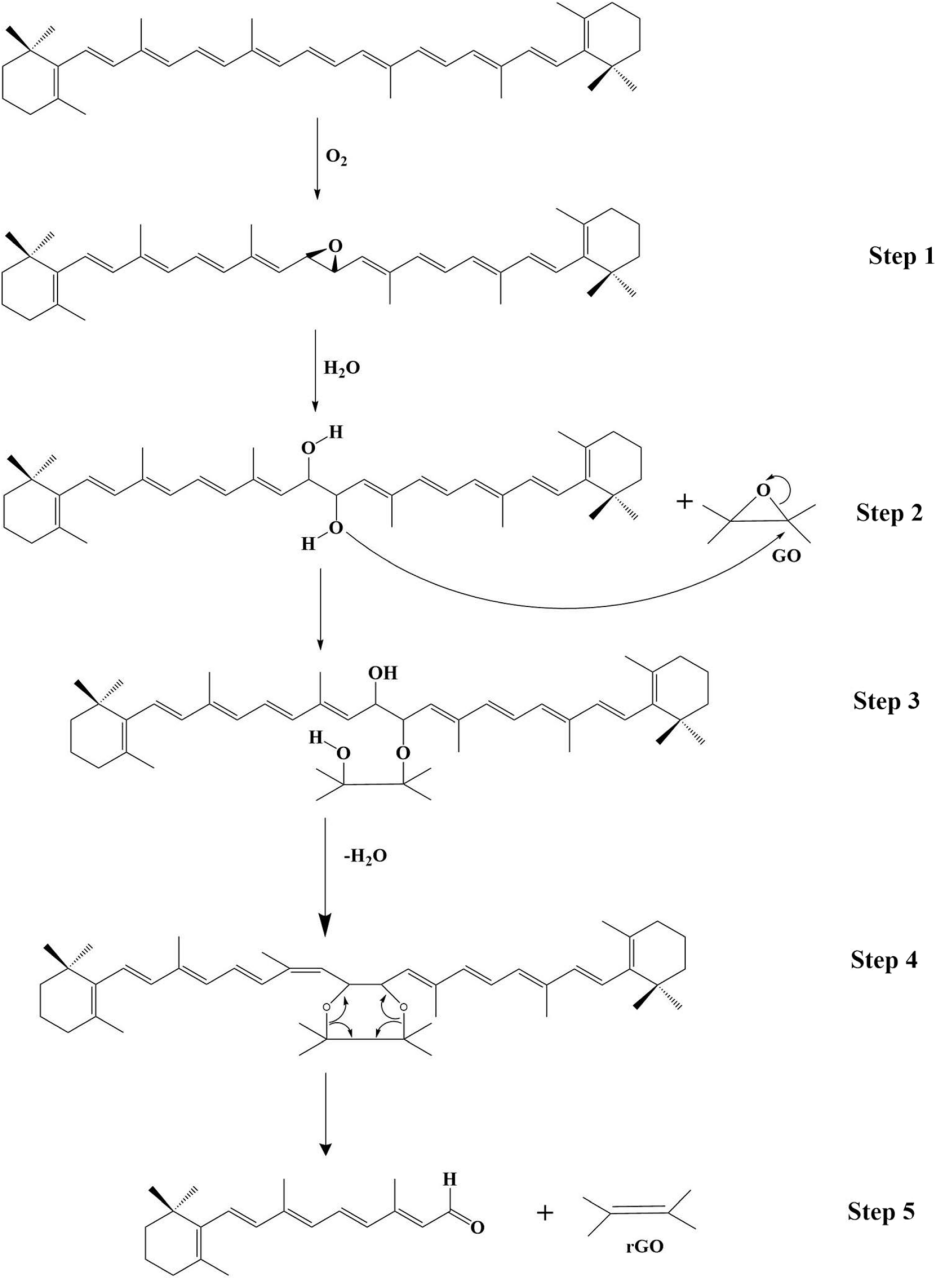
Proposed multi-step reduction mechanism of GO to rGO by β-carotene, involving epoxidation, hydrolysis, and nucleophilic attack. Adapted from ref. 131 (Zaid, *et al.*) with permission under the terms of the Open Access Creative Commons CC-BY-NC-ND license, Elsevier (*Arabian Journal of Chemistry*), copyright 2014.

The reduction mechanisms for biomolecules are specific to their chemical structures. For ascorbic acid, the reduction is believed to proceed *via* a nucleophilic attack on the epoxide groups, similar to that of polyphenols, followed by dehydration.^136,147^ For amino acids containing thiol groups, such as l-cysteine, the thiol group (–SH) is deprotonated and attacks the oxygen functional groups. In this process, the thiol is oxidized to form a disulfide (–S–S–) bond, while GO is reduced to rGO.^148^ For biomolecules with conjugated systems like β-carotene, the proposed mechanism involves the formation of an epoxide on β-carotene itself, which is then hydrolyzed to a diol. The resulting oxygen anions act as nucleophiles, deoxygenating GO (Fig. 8).^131^ The ability to propose such specific chemical pathways is a significant advantage of using pure biomolecules, as it allows for a more rational design of the reduction process.

#### 3.3.3 Application-specific properties and outlook

The high degree of reduction and precise surface chemistry achievable with biomolecules make this rGO ideal for electronics and sensing applications.

##### 3.3.3.1 Sensors and electronics

The excellent C/O ratios and high conductivity achieved with reductants like ascorbic acid and humanin (105 S m^−1^)^146^ make this rGO a prime candidate for conductive films and sensors. Bo, *et al.*^40^ used caffeic acid-reduced rGO to fabricate a gas sensor that exhibited rapid, sensitive responses to NO_2_ and NH_3_. The gas-sensing mechanism relies on charge transfer between gas molecules and the rGO surface, a process that requires a highly conductive material with a low defect density. The ability of biomolecules to produce high-quality, electronically active rGO is a key advantage for these applications.

##### 3.3.3.2 Flexible films

The self-polymerization of specific biomolecules, like dopamine, can be exploited to create robust materials. Luo, *et al.*^33^ used dopamine to both reduce GO and serve as a polymeric binder. As the solvent evaporated, the polydopamine-coated rGO sheets self-assembled into a flexible, layer-by-layer film with a tensile strength of 25 MPa and excellent flame-retardant properties. The outcome demonstrates an intelligent synthesis strategy in which the reducing agent serves a dual purpose, leading to a functional macroscopic material.

## 4 Comparative analysis, challenges, and future outlook

While green reduction methods offer a sustainable alternative to conventional techniques, choosing a method involves trade-offs among cost, scalability, reaction efficiency, and the final properties of rGO. A quantitative comparison of these strategies is essential for guiding future research and application-driven synthesis (Table 4).

**Table 4:** A quantitative comparison of green reduction strategies

Criteria	Plant extract-mediated	Microorganism-mediated	Biomolecule-mediated
Reduction efficiency (C/O ratio)	Moderate to high (typical range: 3.8–11.9)	Moderate (typical range: 2.6–5.9)	High (typical range: 3.9–12.5, esp. ascorbic acid)
Reaction time	Fast (0.5–24 h)	Slow (24–168 h)	Moderate (1–24 h)
Typical electrical conductivity (S m^−1^)	Low to moderate (*e.g.*, 0.358 for gallic acid)	Moderate (*e.g.*, 55.32 for *Shewanella*)	Moderate to high (*e.g.*, 105 for humanin)
Scalability	High (simple equipment, abundant materials)	Low (requires bioreactors, sterile conditions)	Moderate (depends on biomolecule cost)
Control over functionalization	Low (complex mixture of phytochemicals)	Moderate (bio-functionalization possible)	High (well-defined reductant molecule)
Cost	Low	High (culture media, incubation energy)	Variable (low for glucose, high for proteins)

### 4.1 Current challenges and specific research gaps

Despite the significant progress, several challenges must be addressed to advance the field:

#### 4.1.1 Standardization of natural extracts

Plant extracts are complex mixtures whose composition can vary with season, geography, and extraction method. A key research gap is the need for analytical techniques to identify and quantify the primary active reducing agents in these extracts, ensuring reproducibility and process control.

#### 4.1.2 Reaction kinetics and mechanism

The kinetics of most green reduction processes are poorly understood. Detailed kinetic studies are needed to optimize reaction times and temperatures. Furthermore, a deeper mechanistic understanding is required to explain why certain reductants (*e.g.*, artemisinin) are far more effective than others.

#### 4.1.3 Scalability and integration

While many methods work well at the lab scale, scaling them for industrial production remains a significant hurdle, especially for microbial processes. Research is needed on continuous-flow reactors and process optimization to bridge the gap between laboratory synthesis and industrial application.

### 4.2 Future outlook

The future of rGO synthesis lies in the rational design of materials for specific applications. Key future directions include:

#### 4.2.1 Application-driven synthesis

Instead of a one-size-fits-all approach, synthesis methods will be tailored to the end-use. For electronics, the focus will be on achieving the highest possible C/O ratio and conductivity using potent biomolecules. For environmental applications, the goal may be to attain partial reduction to retain functional groups for adsorption. For biomedical uses, microbial methods that ensure biocompatibility will be prioritized.

#### 4.2.2 Structural and functional control

Advanced techniques will enable precise control over the number and type of residual functional groups. The incorporation of heteroatoms (*e.g.*, nitrogen, sulfur) during the green reduction process, a technique known as doping, will be explored to tune the electronic and catalytic properties of rGO.

#### 4.2.3 Hybrid methods

Combining the advantages of different techniques, such as a microwave-assisted reduction using a plant extract, could dramatically shorten reaction times while maintaining the benefits of a green process.

#### 4.2.4 Integration with artificial intelligence

Machine learning algorithms could be used to screen vast libraries of natural compounds to predict their reduction potential and to optimize reaction parameters, accelerating the discovery of new, highly efficient green reduction pathways.

## 5 Conclusion

This review critically examines significant progress in the green reduction of graphene oxide using plant extracts, microorganisms, and biomolecules. These environmentally friendly strategies offer sustainable and often low-cost alternatives to conventional methods that rely on hazardous chemicals. By moving beyond a descriptive summary, we have provided a comparative analysis that links the choice of green reductant to the resulting material properties and its ultimate performance in technological applications. The study reveals that while plant extracts offer a scalable and straightforward route, biomolecules provide superior control and higher reduction efficiency, and microorganisms yield products with exceptional biocompatibility. For instance, the use of potent biomolecules, such as ascorbic acid, offers the most promising path to high-conductivity electronics, while microbially reduced rGO is the clear-cut choice for biocompatible medical applications. Substantial challenges remain, particularly in standardizing natural extracts, understanding reaction kinetics, and scaling up production. However, the immense biodiversity of the natural world offers a vast, largely untapped resource for discovering novel reducing agents. Future research should focus on a more rational, application-driven approach to synthesis, aiming to precisely control the structural and chemical properties of rGO to meet the specific demands of advanced applications in energy, environmental science, and medicine. By addressing current research gaps, green-synthesized rGO is poised to become a cornerstone material in the development of next-generation sustainable technologies.

## Consent to publish

All authors agreed with the manuscript's content and gave explicit permission to publish the work.

## Conflicts of interest

The authors declare that they have no known competing financial interests or personal relationships that could have appeared to influence the work reported in this paper.

## Data Availability

No primary research results, software or code have been included and no new data were generated or analysed as part of this review.
